# Air Pollution: Another Threat to HDL Function

**DOI:** 10.3390/ijms24010317

**Published:** 2022-12-24

**Authors:** Alice Ossoli, Federica Cetti, Monica Gomaraschi

**Affiliations:** Centro Enrica Grossi Paoletti, Dipartimento di Scienze Farmacologiche e Biomolecolari, Università degli Studi di Milano, 20133 Milan, Italy

**Keywords:** high density lipoproteins, air pollution, HDL dysfunction, inflammation, oxidative stress

## Abstract

Epidemiological studies have consistently demonstrated a positive association between exposure to air pollutants and the incidence of cardiovascular disease, with the strongest evidence for particles with a diameter < 2.5 μm (PM_2.5_). Therefore, air pollution has been included among the modifiable risk factor for cardiovascular outcomes as cardiovascular mortality, acute coronary syndrome, stroke, heart failure, and arrhythmias. Interestingly, the adverse effects of air pollution are more pronounced at higher levels of exposure but were also shown in countries with low levels of air pollution, indicating no apparent safe threshold. It is generally believed that exposure to air pollution in the long-term can accelerate atherosclerosis progression by promoting dyslipidemia, hypertension, and other metabolic disorders due to systemic inflammation and oxidative stress. Regarding high density lipoproteins (HDL), the impact of air pollution on plasma HDL-cholesterol levels is still debated, but there is accumulating evidence that HDL function can be impaired. In particular, the exposure to air pollution has been variably associated with a reduction in their cholesterol efflux capacity, antioxidant and anti-inflammatory potential, and ability to promote the release of nitric oxide. Further studies are needed to fully address the impact of various air pollutants on HDL functions and to elucidate the mechanisms responsible for HDL dysfunction.

## 1. Air Pollution as a Cause of Cardiovascular Diseases

The term “air pollution” identifies a complex mixture of particles, gases, and chemicals whose composition varies according to the source and the atmospheric conditions. Based on particle aerodynamic diameter, inhalable air particulate matter (PM) is classified into PM_10_ (coarse, diameter < 10 μm), PM_2.5_ (fine, diameter < 2.5 μm), and nanoparticles (ultrafine, UFP, diameter < 0.1 μm). Gaseous pollutants include nitrogen oxides (NO_x_), sulphur dioxide (SO_2_), carbon monoxide (CO), ozone (O_3_), and volatile organic compounds. Metals, toxic agents, pathogens, hydrocarbons, and other organic compounds may be also carried by PM [1,2]. Air pollutants are primarily generated by the combustion of fossil fuels and erosion or by non-combustion sources as agriculture. In urban areas, fine and ultrafine particles are released by vehicles, industrial plants and domestic heating together with nitrogen oxides. Once released in the atmosphere, these particles are modified into the so-called secondary pollutants by the action of water vapor and sunlight and the inclusion of other chemicals, as metals [3].

Evidence linking the exposure to air pollution to the incidence of cardiovascular (CV) events arise mainly from PM_2.5_ measurements. Indeed, PM_2.5_ is considered the most harmful fraction of air pollution and the fifth risk factor for mortality in 2015 [4]. This is likely because PM_2.5_ is relatively stable in the atmosphere, thus facilitating its reliable detection by monitoring stations in exposure studies. On the contrary, nanoparticles are short-lived and, consequently, their presence could not be adequately assessed by monitoring stations [2]. Interestingly, while the levels of PM_10_ and PM_2.5_ have fallen in many countries worldwide, a concomitant increase in nanoparticles was observed. These ultrafine particles are particularly harmful due to their small size and their large surface area, thus allowing them (i) to deeply penetrate the respiratory tract and potentially the bloodstream, and (ii) to react with and carry many chemicals which could increase their oxidative potential [2,5].

**Figure 1:**
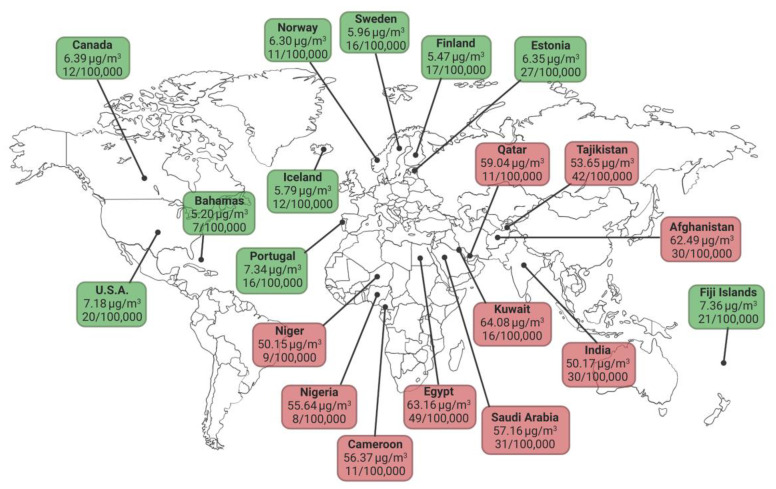
WHO estimates of air pollution and death rates for ischemic heart disease in 2019.

In spite of variations in the assessment of air pollution, study design and enrolled populations, a large number of epidemiological studies have consistently demonstrated a positive association between the exposure to air pollutants and the incidence of cardiovascular diseases (CVD). According to the Global Burden of Disease, air pollution accounted for 19% of all cardiovascular deaths in 2015 [4]. As stated above, the strongest evidence is for PM_2.5_ and both the American Heart Association (AHA) and the European Society of Cardiology (ESC) included PM_2.5_ among the modifiable risk factor for cardiovascular outcomes as cardiovascular mortality, acute coronary syndrome, stroke, heart failure, and arrhythmias (Figure 1) [2,6]. In a recent estimate of the AHA, the risk of CV events increases by 1–2% for short-term exposures and by 5–10% for long-term exposures every 10 μg/m^3^ of PM_2.5_, causing the 13.3% of all cardiovascular deaths [7]. On the same line, in a large cohort study performed in Europe each 5 μg/m^3^ increase in long-term PM_2.5_ exposure was associated with a 18% increase in nonfatal acute coronary events [8]. Interestingly, adverse effects of air pollution are surely more pronounced at higher levels of exposure but were also shown in countries with low levels of air pollution [9], indicating no apparent safe threshold (Figure 1). Moreover, air pollution seems particularly harmful in elderly subjects and in those with pre-existing CVD, elevated body mass index or diabetes [10]. 

Levels of PM_2.5_ in the ten less polluted countries (green tags) and the ten more polluted countries (red tags), according to the World Health Organization (WHO) Observatory data. For each country the air pollution attributable death rate (expressed as number of deaths per 100,000 population per year) for ischemic heart disease is also indicated. Created with BioRender.

**Figure 2:**
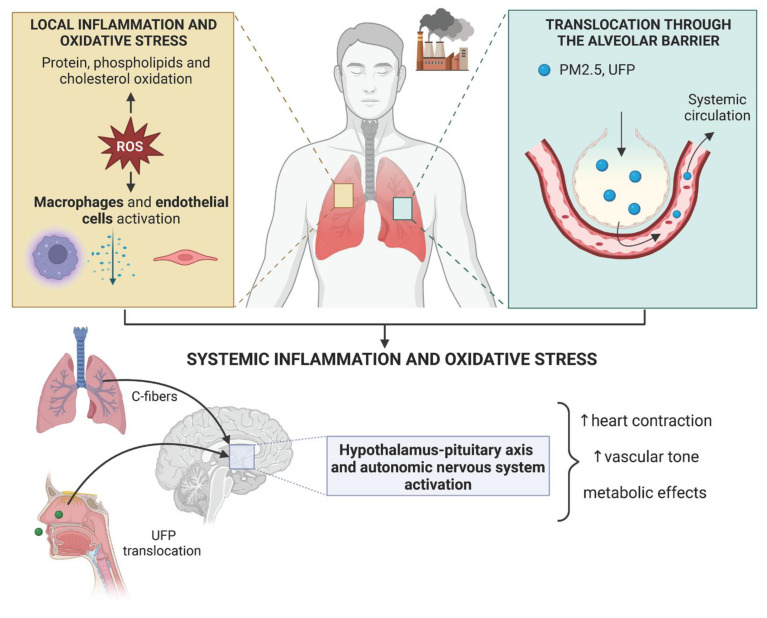
Damaging mechanisms of air pollution on the cardiovascular system.

It is generally believed that short-term exposure to air pollution can lead to acute CV events by destabilizing vulnerable plaques, while long-term exposure can accelerate atherosclerosis progression by promoting dyslipidemia, hypertension, and other metabolic disorders [1] (Figure 2). Thus, a synergistic effect of cumulative exposure to PM_2.5_ and traditional CV risk factors is proposed. However, it is difficult to separate the effect of short-term and long-term exposures to air pollution, since people exposed to high levels of air pollution for short periods of time are frequently exposed to chronically elevated levels as well.

The inhalation of air pollutants causes the local production of reactive oxygen species (ROS) and inflammatory molecules in the lungs through the activation of alveolar macrophages and endothelial cells. The progression towards a systemic inflammatory state is due to (i) the release of locally produced soluble inflammatory mediators in the circulation and (ii) the direct translocation of PM_2.5_ and ultrafine particles (UFP) in the systemic circulation across the alveolar barrier. Air pollution also activates the hypothalamus-pituitary axis and the autonomic nervous system through (i) the activation of C-fibers in the lungs and (ii) the direct translocation of UFP in the central nervous system. Created with BioRender.

## 2. Mechanisms of Action

The mechanisms responsible for the increased cardiovascular risk due to air pollution can be summarized into: (i) local responses in the lungs which include oxidative stress, pulmonary inflammation, and ion channel/receptor activation; (ii) direct or indirect systemic activation of signaling pathways due to the generation of biologic intermediates, autonomic nervous system imbalance, and particle translocation; and (iii) end-organ effector mechanisms, as endothelial dysfunction, characterized by vasoconstriction, thrombosis and loss of barrier integrity, a key event in atherogenesis [11] (Figure 2). 

The inhalation of air PM in the lungs causes local oxidative stress, which is enhanced by the presence of highly reactive oxidant gases such as O_3_ and NO_2_ [12,13]. The overproduction of reactive oxygen species (ROS), especially superoxide, can be related to the PM-mediated activation of nicotinamide adenine dinucleotide phosphate oxidases (NADPH oxidase), mitochondrial enzymes, and uncoupled nitric oxide (NO) synthase, with a concomitant progressive depletion of antioxidant defenses as superoxide dismutase (SOD) [14,15,16]. Generated ROS lead to the oxidation of proteins and lipids in the surfactant milieu, in endothelial cells and in alveolar macrophages, which results in the induction of inflammation through activation of Toll-like receptor (TLR) signaling [17]. In addition, the presence of polycyclic hydrocarbons (PAH) on PM can activate the aryl hydrocarbon receptors (AhR), which, in turn, trigger pro-inflammatory nuclear factor-kappa (NF-kB) and nuclear factor erythroid 2–related factor (Nrf2) signaling, that regulates cell resistance to oxidation [18,19].

Oxidized and inflammatory molecules generated in the lungs can reach the systemic circulation with the development of a systemic inflammatory state, thus favoring CVD [20]. Moreover, the transition from a local to a systemic reaction against air pollution can be explained by the particle translocation hypothesis, which is based on the evidence that PM_2.5_ and nanoparticles are small enough to cross the alveolar-capillary membrane and enter the circulation [21] (Figure 2). Indeed, inhaled gold nanoparticles, used as a surrogate for diesel exhaust, can be detected in the blood as well as in the vascular wall, especially in atherosclerotic plaques [22]. Once in the systemic circulation, PM can activate TLR4 signaling and NF-kB activation, increase the expression of cytokines, chemokines, cell adhesion molecules, and induce lipid peroxidation [15,23,24,25]; in the long-term, air PM can promote the senescence of leukocytes via telomere shortening [26]. In addition, air pollution can decrease the availability of NO due to eNOS uncoupling, with a concomitant increase in vasoconstrictor endothelin-1, vascular endothelial growth factor and matrix metalloproteinases 2/9 [27,28,29]. Long-term exposure to air pollution has been also associated with the depletion of endothelial progenitor cells [30,31]. Air PM were shown to affect platelet activation, coagulation, and fibrinolysis, leading to a pro-coagulant and antifibrinolytic state [32] through the increase in interleukin-1β, CD40 ligand, P-selectin, and fibrinogen/fibrin degradation products [33]. Recently, miRNAs were also investigated as mediators of PM damaging effects: PM exposure has been associated with high levels of circulating miRNAs carried by extracellular vesicles [34,35,36] suggesting a role for epigenetics in PM-induced CVD [37]. 

Finally, the exposure to air pollution stimulates the activation of the hypothalamus-pituitary axis (HPA) axis and of the autonomic nervous system, which can increase blood pressure, modulate heart rate, reduce cardiac function, and negatively affect metabolic control [38] (Figure 2). Indeed, air PM can activate the C-fibers in the lungs by interacting with the transient receptor potential cation channel subfamily A member 1 (TRPA1) on airway sensory neurons resulting in neurogenic inflammation [39]. Moreover, neurogenic inflammation can be directly triggered by nanoparticles that can penetrate the central nervous system [40]. The activation of the autonomic nervous system was demonstrated by the increased urinary excretion of catecholamines, including epinephrine, norepinephrine, and dopamine after long-term exposure to air pollutants [41]. PM-mediated HPA activation promotes the release of corticotropin-releasing hormone (CRH), adrenocorticotropic hormone (ACTH), and cortisol in the circulation, with a consequent increase in sodium and water retention [42,43,44]. PM exposure also promotes the overexpression of α2B adrenergic receptor (Adra2b) in the brain and in macrophages. In the brain, the activation of Adra2b leads to anxiety and behavioral changes besides causing blood pressure elevation [45]. In macrophages, the activation of Adra2b leads to pulmonary and systemic inflammation and even thrombosis [46]. 

Overall, the local and systemic effects of air pollutants described above can explain their negative impact of the cardiovascular system, thus increasing the risk of CVD.

## 3. Effects of Air Pollution on HDL-Cholesterol Levels

Whether short- or long-term exposure to air pollution is associated with changes in plasma levels of lipids and lipoproteins is still debated, especially regarding high density lipoprotein-cholesterol (HDL-C) for which negative, positive or no associations were described [47,48,49,50,51,52,53,54]. Again, differences in exposure assessment, study design, enrolled population and geographic localization, together with the impact of possible confounding factors, could be responsible for the inconsistency among studies. Overall, no association between HDL-C levels and short-term exposure to air pollutants was observed [47]. Regarding long-term exposure, some studies found a positive association with total and low-density lipoprotein (LDL) cholesterol levels, and a negative one with HDL-C [47,48,49]. For example, in the longitudinal Study of Women’s Health Across the Nation performed in the US, a 0.7% decrease in plasma HDL-C levels and 0.6% decrease in apoA-I were calculated for each 3 μg/m^3^ increase in one-year PM_2.5_ exposure [47]. Consistently, in the longitudinal MESA-Air study performed in a multiethnic US cohort free of cardiovascular disease, higher exposures to PM_2.5_ or black carbon were associated with lower HDL particle numbers or lower HDL-C levels. Interestingly, no modifying effect of age, ethnicity, smoking, obesity, and diabetes was observed [48]. A cross-sectional study performed in 33 Chinese communities confirmed the positive association of PM_2.5_ with total and LDL cholesterol, and a negative one with HDL-C levels. In particular, each 10 μg/m^3^ increment of PM_2.5_ resulted in a 1.1% decrease in HDL-C [49]. On the contrary, no associations were found in two large European cohorts (HUNT3 in Norway and Lifelines in the Netherlands) and in a nationwide US study [50].

Few studies addressed the impact of air pollution on plasma levels of lipids in children, which are considered particularly susceptible due to lung development and the time spent outdoor. Results are again conflicting. The US Children’s Health and Air Pollution Study was performed in a highly polluted area and highlighted a decrease in HDL-C with increasing exposure to multiple air pollutants [51]. However, this association was not detected in an Italian or in a Chinese cohort [52,53]. The authors pointed at a different incidence of obesity between the cohorts, with the 48.7% of overweight/obese participants in the US one.

Lastly, the effects of air quality improvement on the lipid profile are unknown. Recently, a survey conducted in China evaluated plasma lipids before and after the implementation of clear air actions. Even after adjustment for different covariates, total and LDL cholesterol were significantly reduced when PM_2.5_ levels decreased, but no association was found for HDL-C [54].

## 4. Cardiovascular Protection by HDL

The inverse association between plasma levels of HDL-C and atherosclerotic CVD is well established and HDL-C levels are included in the ASCVD Pooled Cohort Equations and in the European SCORE (Systematic COronary Risk Evaluation) risk charts [55]. However, drugs able to increase HDL-C, such as fibrates, niacin, and cholesteryl transfer protein inhibitors, do not demonstrate consistently improved atherosclerotic CVD outcomes [56,57,58,59], supporting the hypothesis that HDL-C level is not a good predictor of HDL-mediated atheroprotective potential. HDL are a highly heterogeneous class of lipoproteins with varying size, shape, and protein/lipid composition. This heterogeneity is the consequence of the complexity of their metabolism and interconversion within the plasma compartment, where it is possible to identify different HDL subclasses [60]. Small discoidal HDL, called preβ-HDL, are mainly generated by the lipidation of the main protein component, apolipoprotein A-I (apoA-I), through the interaction with the ATP-binding cassette transporter A1 (ABCA1) [61]. These particles are good acceptors of additional cholesterol through ABCA1 and become substrate of lecithin:cholesterol acyltransferase (LCAT), the enzyme responsible for cholesterol esterification in the plasma compartment, which lead the maturation of discoidal HDL into spherical particles with cholesteryl esters (CE) in the core [62] (Figure 2). Spherical HDL can interact with cholesteryl ester transfer protein (CETP), which promotes the exchange of CE and triglycerides (TG) between HDL and apoB-containing lipoproteins, thus modifying the lipid core of HDL and generating larger and less dense particles. Finally, the action of different lipases, as endothelial lipase (EL) or phospholipid transfer protein (PLTP), leads to the hydrolysis of TG and phospholipids (PL) with the regeneration of small lipid poor HDL [63]. The major site of apoA-I and small HDL clearance is the kidney, where they are removed from the circulation by glomerular filtration [64]. The distribution of HDL into subclasses is relevant, since different subclasses may exert distinct atheroprotective functions, and their prevalence in plasma could be not related with the levels of HDL-C.

**Figure 3:**
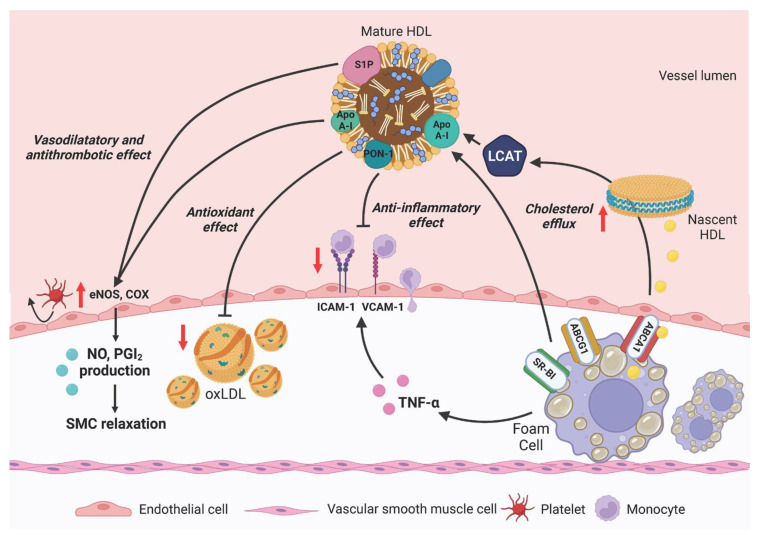
Atheroprotective functions of HDL on the vascular wall.

The best characterized atheroprotective function of HDL is their ability to accept cholesterol from peripheral tissues (Figure 3). Cholesterol efflux towards HDL represents the first and rate-limiting step of the so-called reverse cholesterol transport (RCT), through which cholesterol is routed to the liver for excretion. Cholesterol efflux occurs through passive diffusion, facilitated diffusion, or active transport by the interaction of HDL with different transporters and receptors on the cell membrane. Lipid-free apoA-I and discoidal preβ-HDL are the best acceptors of cholesterol through ABCA1, while spherical HDL preferentially interact with ABCG1 and with the scavenger receptor SR-BI [65]. The cholesterol efflux capacity (CEC) of HDL is used to estimate the efficiency of the entire RCT process in humans and it is inversely correlated with individual cardiovascular risk regardless of plasma HDL-C levels [66]. HDL can also contribute to atheroprotection by exerting antioxidant and anti-inflammatory activities, and by preventing and correcting the endothelial dysfunction, a key event in the atherosclerotic process [67] (Figure 3). In particular, HDL are able to modulate endothelial function by promoting vasorelaxation and inhibiting platelet adhesion, which is mediated by the increase in nitric oxide (NO) and prostacyclin production by endothelial cells (ECs). In addition, HDL can prevent the production of pro-inflammatory and cell adhesion molecules by endothelial and circulating cells, promote EC migration and proliferation, while inhibiting cell apoptosis [68]. HDL inhibit LDL oxidation, either by limiting lipid peroxidation and by taking up lipid hydroperoxides from other lipoproteins through their protein and lipid cargo [69]. In recent years, a relevant role of HDL in innate and adaptive immunity has been highlighted [70]. It has been demonstrated that both apolipoproteins and specific HDL-associated lipids, such as apoA-I and sphingosine 1-phosphate (S1P), are able to modulate the complement system and other signaling pathways involved in innate and adaptive immunity [70]. In particular, HDL are involved in the polarization of macrophages to proinflammatory M1 or anti-inflammatory M2 phenotype, while HDL-associated S1P, through the modulation of receptors located in the lipid rafts, regulates the activity and the differentiation of circulating lymphocytes. As stated above, some HDL subclasses are more effective than others in mediating particular protective effects [60]; for example, small HDL have higher capacity to prevent LDL oxidation and the production of cell adhesion molecules by ECs than other HDL subclasses [71,72,73].

High-density lipoproteins (HDL) are the main acceptors of cholesterol from peripheral cells through the interaction with the ATP-binding cassette transporters A1 and G1 (ABCA1 and ABCG1), and the scavenger receptor type BI (SR-BI). Cholesterol within HDL is then esterified by lecithin:cholesterol acyltransferase (LCAT). Through their protein and lipid cargo HDL can inhibit the oxidation of low-density lipoproteins (LDL). HDL are also able to inhibit the cytokine-induced production of cell adhesion molecules (as ICAM-1 and VCAM-1) by endothelial cells, thus limiting the adhesion and extravasation of monocytes. HDL promote the release of nitric oxide (NO) and prostacyclin (PGI_2_) by endothelial cells, which inhibit the adhesion of platelets and induce the relaxation of smooth muscle cells (SMC). Created with BioRender.

In the last twenty years, several cell-based and cell-free functional assays have been developed and used to assess HDL atheroprotective activities in the contest of different physiological and pathological states, or after dietary/pharmacological interventions. The most widely used are CEC of apoB-depleted plasma, HDL ability to prevent LDL oxidation and to preserve EC homeostasis (evaluated as production of NO, inhibition of the expression of cytokines and adhesion molecules, promotion of cell migration and proliferation, inhibition of apoptosis, etc.) [74]. These assays allowed the demonstration that different pathologic conditions, especially those associated with acute or chronic inflammation, significantly impair HDL atheroprotective functions. In these conditions, the protein and lipid cargo of HDL is altered with a consequent negative impact on HDL function [75]. For example, HDL proteome is characterized by the presence of the acute-phase protein serum amyloid A (SAA), which displaces apoA-I from HDL, or by the chemical modification of HDL-associated proteins, as oxidation and glycation [75]. In addition, in patients with autoimmune diseases, as systemic lupus erythematosus and rheumatoid arthritis, the presence of autoantibodies against apoA-I has been described and associated with an impairment of HDL function, likely contributing to the increased CV risk of these patients [76]. 

## 5. Effects of Air Pollution on HDL Function

**Figure 4:**
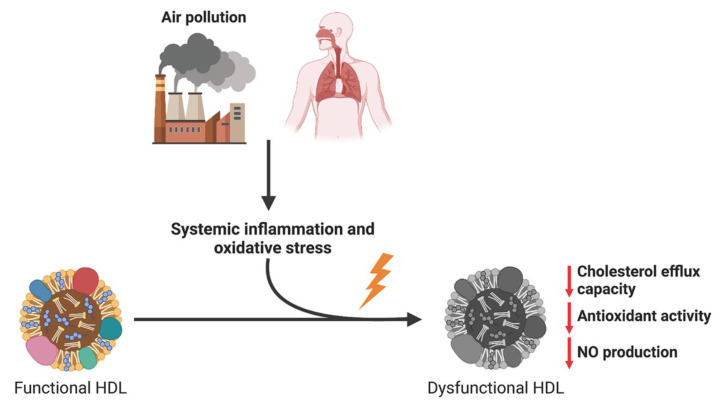
Effects of air pollution on HDL function.

In view of the recent hypothesis that HDL function could be more relevant than HDL levels (at least when measured as their cholesterol content) for atheroprotection, a handful of studies have been already performed to investigate the impact of air pollution on HDL properties [24,25,77,78,79,80,81,82,83,84]. To date, HDL ability to prevent oxidation, and to promote cholesterol efflux from macrophages and NO release from endothelial cells were investigated (Figure 4). Three cross-sectional studies addressed the association between HDL function and the exposure to air pollutants during the week before blood collection through data from air monitoring stations and/or personal samplers. The first one was performed in 50 healthy adults living in southeast Michigan (USA); on average, the exposure to PM_2.5_ during the 7 days before sample collection was below the annual National Ambient Air Quality Standards. Nevertheless, higher levels of PM_2.5_ were associated with a significant decrease in HDL-mediated cholesterol efflux, especially at lag days 5 and 6: for each 10 μg/m^3^ increase in PM_2.5_ cholesterol efflux decreased by 1.93% and 1.62%, respectively [77]. These results suggest that the damage induced by PM_2.5_ takes several days to become evident in term of impaired efflux capacity of HDL. The second study was performed in China in 73 healthy adults exposed to high levels of air pollutants, as PM_2.5_, UFP, black carbon, CO, NO_2_, and SO_2_ [78]. The detrimental effect of air pollutants on HDL ability to promote cell cholesterol efflux was even more pronounced, ranging from 2.3% to 5.0% decrease for interquartile increases of PM_2.5_, black carbon, and CO, especially at lag days 3 to 7. The HDL oxidation index (HOI), an in vitro measure of lipid peroxidation, was also assessed: significant increases of HOI were detected for interquartile increases of SO_2_, UFP sized 5–50 nm, and 50–100 nm at lag days 3 to 7. This study confirmed the delay between the exposure to pollution and the impairment of HDL function and suggested that distinct components of air pollution may differentially affect HDL atheroprotective functions. Recently, we further extended the assessment of the impact of pollution on HDL function by evaluating the association between exposure to PM_10_, PM_2.5_, and NO_2_, and HDL ability to promote the release of vasoactive NO by cultured endothelial cells [79]. The study was performed in Lombardy (Italy) with average PM exposure above the annual limits. Since both healthy lean subjects and overweight/obese ones were included, we were able to assess the modifying effect of body weight on the association between PM or NO_2_ exposure and HDL function. Interestingly, in lean subjects, a positive association between HDL function and exposure to PM_10_ and PM_2.5_ at lag day 1 was found, indicating a compensatory response of the HDL system to air pollution-induced damaging effects. However, this association was progressively lost at increasing body weight, with a tendency towards a negative association in obese subjects. These results are consistent with an in vitro study showing that HDL inhibited the harmful effects of diesel exhaust particles on endothelial cells and macrophages, while dysfunctional HDL did not [80]. Moreover, the impairment of HDL function caused by the exposure to air pollution or by coronary heart disease was similar. The reduction in HDL ability to promote the release of NO occurred earlier than the previous studies (lag day 1). The timing is consistent with a previous study showing that an acute exposure to diesel exhaust can impair vascular function for the following 24 h, likely due to a reduced availability of nitric oxide [81]. 

The exposure to air pollution has been associated with a reduction in HDL atheroprotective functions, as their cholesterol efflux capacity, antioxidant activity and ability to promote the release of nitric oxide by endothelial cells. The mechanisms responsible for HDL dysfunction in subjects exposed to air pollution have not been fully elucidated yet but could be related to the systemic pro-oxidant and inflammatory state triggered by pollutants. Created with BioRender.

The observational nature of cross-sectional studies makes difficult to imply a causal link between air pollution and HDL dysfunction due to the presence of several confounding factors. However, evidence from two controlled exposure studies in humans is also available. In the first study, healthy subjects underwent the inhalation of filtered air or of high levels of coarse PM from a rural source for 2 h with a double-blind crossover design and HDL function was assessed 2 h and 20 h after inhalation. HDL-mediated cholesterol efflux and HDL oxidation index were not affected by coarse PM [82]. In the second study, healthy subjects were exposed to filtered air or to very high levels of PM_2.5_ for 2 h, for a total of 4 exposures at least 2 weeks apart. HDL oxidation index was measured before, as well as 1 h and 20 h after each exposure, and a transient and modest increase in HOI was observed only in subjects with a normal HOI at baseline [83]. Thus, a confirmation of pollution-induced HDL dysfunction in humans is still lacking. Controlled exposure studies were also performed in animal models of atherosclerosis, as the LDL-receptor (*ldlr*) and the apolipoprotein E (*apoE*) knock-out mice. In *apoE*-KO mice, the exposure to UFP for 75 h over a 40-day interval caused a significant increase in atherosclerotic lesions and an impairment of HDL anti-inflammatory activity, measured as their ability to prevent LDL-induced chemotaxis. The effects of PM_2.5_ were less evident [24]. *ApoE*-KO mice were also exposed to diesel exhaust, which is enriched in UFP, for 2 weeks [25]. The exposure to diesel exhaust dramatically affected HDL function, since they completely lost their anti-inflammatory and antioxidant properties, even causing an increase in LDL-induced monocyte migration and of oxidation. On the contrary, HDL ability to promote cell cholesterol efflux was not affected. Interestingly, HDL function was still partially impaired one week after the exposure. The detrimental effect of UFP on atherosclerosis progression and on the antioxidant properties of HDL was confirmed in *ldlr*-KO mice fed a high-fat diet and exposed to UFP for 5 h/day, 3 days/week, for a total of 10 weeks [84]. Compared to mice exposed to filtered air, *ldlr*-KO mice showed a significant increase in HDL oxidation index and of atherosclerotic lesion area. Interestingly, the phenotype was attenuated when *ldlr*-KO mice were treated with the apoA-I mimetic peptide D-4F.

The mechanisms responsible for HDL dysfunction induced by air pollutants have not been elucidated yet. HDL could be modified as in other pathologic conditions characterized by an acute or chronic systemic inflammatory response (Figure 3); indeed, in exposure studies, dysfunctional HDL were associated with elevated plasma levels of inflammatory markers, as C-reactive protein, TNFα, or 8-isoprostanes [25,78]. Pollution-induced increase in oxidative stress could also affect HDL function, likely promoting the peroxidation of HDL lipids more than oxidative changes in HDL proteins [25,77]. These alterations could result in an impairment of HDL antioxidant potential, due to an increased content of lipid hydroperoxides and to a reduced activity of antioxidant enzymes such as paraoxonase-1 or LCAT [24,25,82]. Regarding HDL ability to induce NO release by endothelial cells, in our observational study, changes in HDL activity were not related to circulating markers of inflammation or oxidative stress [79], leaving this issue completely open. Since air pollution can exacerbate autoimmune diseases [85,86], HDL dysfunction could also be due to the presence of autoantibodies against apoA-I; this intriguing hypothesis has not been formally investigated yet.

## 6. Conclusions and Perspectives

Air pollution is now considered a modifiable risk factor for atherosclerotic cardiovascular disease. Oxidative stress and inflammation triggered by air pollutants can be responsible for the development and progression of atherosclerosis in exposed subjects by directly damaging the arterial wall and/or by favoring the development of other pro-atherogenic conditions, as hypertension and dyslipidemias. On this line, recent evidence suggests a detrimental effect of air pollution on HDL function, which can contribute to atherosclerosis progression beyond the possible modulation of plasma HDL-C levels. Larger and controlled studies are needed to fully address this issue and to better clarify which HDL activities are mainly compromised, the timeframe of exposure and of HDL functional recovery, the interactions between risk factors, and the mechanisms responsible for HDL dysfunction. Moreover, the efficacy of possible preventive or mitigation strategies is totally unknown.

Overall, the HDL system, which exerts key protective activities to maintain vessel wall integrity, is sensitive to the detrimental effects of several pathophysiological conditions, and air pollution seems to be the latest name on the list.
